# Oligosaccharides, Gut Microbiota, and Mucosal Health: Molecular Mechanisms and Implications for Early-Life Gut Health

**DOI:** 10.3390/nu18203369

**Published:** 2026-10-14

**Authors:** Donatella Mentino, Giovanni Scillitani, Daniela Semeraro, Marco Vito Guglielmi, Nastasia Taldone, Michelle Laterza, Rossella Vitale, Maria Felicia Faienza, Maria Mastrodonato

**Affiliations:** 1 Department of Biosciences, Biotechnology and Environment, University of Bari Aldo Moro, 70125 Bari, Italy; giovanni.scillitani@uniba.it (G.S.); daniela.semeraro@uniba.it (D.S.); marco.guglielmi@uniba.it (M.V.G.); n.taldone@alumni.uniba.it (N.T.); m.laterza55@studenti.uniba.it (M.L.); maria.mastrodonato@uniba.it (M.M.); 2 Pediatric Unit, Department of Precision and Regenerative Medicine and Ionian Area, University of Bari Aldo Moro, 70124 Bari, Italy; r.vitale7@phd.uniba.it (R.V.); mariafelicia.faienza@uniba.it (M.F.F.)

**Keywords:** oligosaccharides, human milk oligosaccharides, gut microbiota, intestinal mucus, mucosal barrier, bacterial adhesion, pediatric nutrition

## Abstract

Oligosaccharides are structurally diverse glycans that influence intestinal homeostasis through mechanisms extending beyond conventional prebiotic activity. This review examines how dietary, human milk, host-derived, and biotechnologically produced oligosaccharides modulate the gut microbiota and mucosal host–microbe interactions, with particular attention to pediatric health. Non-digestible oligosaccharides support saccharolytic microbial communities, cross-feeding networks, and short-chain fatty acid production, thereby affecting luminal pH, epithelial metabolism, mucus production, barrier integrity, and immune signaling. Their biological effects are strongly determined by monosaccharide composition, linkage configuration, branching, charge, degree of polymerization, terminal residues, and spatial presentation. Neutral human milk oligosaccharides and galacto-oligosaccharides may reduce pathogen colonization by mimicking host glycans, occupying adhesin-binding sites, modifying epithelial glycosylation, and limiting biofilm initiation. Selected chitooligosaccharides can exert direct antibacterial effects through charge-dependent interactions with bacterial envelopes, although these effects depend on molecular size, charge, and chemical derivatization. In addition, the α1,4-GlcNAc-terminated O-glycan motif of gastric MUC6 provides a distinct example of host-associated glycan-mediated inhibition of bacterial metabolism. During infancy, human milk oligosaccharides promote a *Bifidobacterium*-enriched microbiota and may contribute to intestinal and immune maturation. Galacto- and fructo-oligosaccharide-supplemented formulas reproduce some microbiota- and metabolite-related effects, although evidence for preventing infectious, allergic, or inflammatory disease remains inconsistent. Experimental findings also suggest a role in recovery from antibiotic-associated dysbiosis, but clinical evidence is limited by heterogeneous populations, exposures, formulations, and endpoints. Future studies should use structurally defined compounds, mechanism-specific assays, and clinically relevant outcomes to establish causal structure–function relationships and identify pediatric populations most likely to benefit.

## 1. Introduction

Oligosaccharides are structurally diverse carbohydrates composed of a limited number of monosaccharide residues linked by glycosidic bonds. Although their precise boundaries vary across chemical and nutritional classifications, they are generally positioned between simple sugars and polysaccharides and commonly contain approximately 2–10 monosaccharide units, with some functional definitions extending to 20 units. Their biological activity depends not only on degree of polymerization but also on monosaccharide composition, linkage configuration, branching, charge, terminal residues, and resistance to digestion by host enzymes. Accordingly, oligosaccharides of similar size may display markedly different physicochemical and biological properties. Major classes include fructo-oligosaccharides and oligofructose, galacto-oligosaccharides, xylo-oligosaccharides, arabino-oligosaccharides, mannooligosaccharides, isomalto-oligosaccharides, pectic oligosaccharides, chitin- and chitosan-derived oligosaccharides, and human milk oligosaccharides. HMOs (Human Milk Oligosaccharides) form a particularly complex group of unconjugated glycans built on a lactose core and extended with galactose, N-acetylglucosamine, fucose, and/or sialic acid. They are commonly classified as neutral non-fucosylated, neutral fucosylated, or acidic sialylated structures [1,2]. Importantly, the terms oligosaccharide and prebiotic are not synonymous. Oligosaccharide denotes a chemical category, whereas a prebiotic is a substrate selectively utilized by host microorganisms that confers a health benefit [1]. Oligosaccharides can influence intestinal homeostasis through mechanisms that extend beyond their conventional prebiotic activity. Non-digestible oligosaccharides may be selectively metabolized by members of the gut microbiota, promoting microbial cross-feeding and the production of biologically active metabolites. Other oligosaccharides can interact with the intestinal mucus layer and epithelial glycome, mimic host–cell glycan receptors, inhibit bacterial adhesion, affect biofilm formation, or modulate epithelial and immune responses. Selected charged structures may additionally alter bacterial-envelope integrity or interfere with specific metabolic pathways. These activities are frequently grouped under broad terms such as “prebiotic”, “antibacterial”, or “immunomodulatory”, although they represent mechanistically distinct biological outcomes. In particular, reduced adhesion, ecological exclusion, metabolic inhibition, membrane permeabilization, and bacterial killing should not be considered equivalent. Despite the expanding literature on bioactive oligosaccharides, several knowledge gaps remain. First, mechanistically distinct effects are frequently grouped under broad labels such as prebiotic, antibacterial, or immunomodulatory activity. Second, structure–function relationships are not consistently integrated across microbial, mucosal, and host pathways, limiting extrapolation between chemically distinct oligosaccharides. Third, pediatric evidence spans in vitro models, animal studies, observational cohorts, and clinical interventions using heterogeneous compounds, formulations, and endpoints, making translation to clinically meaningful outcomes challenging. Early life represents a particularly relevant context because the gut microbiota, mucus barrier, epithelial glycome, and immune system are still undergoing maturation, while exposure to milk-derived and supplemented oligosaccharides is particularly prominent during this developmental period. This review addresses these gaps by distinguishing molecular mechanisms, linking structural features to biological effects, and critically integrating mechanistic evidence with early-life and pediatric applications.

The primary objective of this review is to provide an integrated and critical analysis of the mechanisms through which dietary, milk-derived, and biotechnologically produced oligosaccharides, together with selected host-associated glycan motifs used as mechanistic comparators, modulate the gut microbiota and host–microbe interactions at the intestinal mucosa. A central aim is to distinguish effects that are frequently grouped together, namely microbiota-mediated, host-mediated, anti-adhesive, antibiofilm, and bactericidal activities—despite involving substantially different molecular and biological pathways. Particular attention is given to the relationships among oligosaccharide structure, microbial utilization, mucus and glycome remodeling, bacterial-surface interactions, epithelial and immune responses, and biofilm development. The review further aims to bridge mechanistic evidence with pediatric nutrition by examining the roles of human milk oligosaccharides and supplementation with galacto-oligosaccharides (GOS) and fructo-oligosaccharides (FOS) in early microbial colonization, intestinal and immune maturation, and recovery from antibiotic-associated dysbiosis. By integrating evidence from glycobiology, microbial ecology, nutrition, and pediatric research, this review seeks to define the current state of knowledge, identify the principal methodological and translational limitations, and establish priorities for the development of evidence-based oligosaccharide interventions. This article is intended as a narrative review and does not follow a formal systematic-review or study-selection protocol.

## 2. Relevance to the Gut Microbiota and Human Health

Non-digestible oligosaccharides can reach the colon and be metabolized by bacterial carbohydrate-transport and degradation systems. Their utilization may favor saccharolytic microorganisms, including selected *Bifidobacterium* and *Lactobacillus* species, while also supporting broader microbial cross-feeding networks. Primary degraders can release smaller carbohydrates or metabolites that are subsequently consumed by other community members. Consequently, oligosaccharide-mediated effects cannot be reduced to changes in one or two bacterial genera but should also be evaluated at the level of microbial function and ecological interactions [3,4]. Microbial fermentation generates the major intestinal SCFAs acetate, propionate, and butyrate, as well as other fermentation products such as lactate. Lactate is an important cross-feeding intermediate and can be utilized by selected microbial populations for the production of propionate or butyrate. Butyrate is an important energy source for colonocytes and contributes to epithelial homeostasis, whereas acetate can also participate in microbial cross-feeding networks. Short-chain fatty acids (SCFAs) also influence luminal pH, epithelial metabolism, mucin expression, and immune signaling. These mechanisms help explain why oligosaccharides may support intestinal health through combined effects on microbial composition, metabolite production, barrier function, and colonization resistance [4,5,6]. Dietary context is a major determinant of these responses. Plant-rich diets containing fermentable carbohydrates generally promote saccharolytic metabolism, whereas Western dietary patterns characterized by high fat, high sugar, and low fiber can favor bile-tolerant microorganisms, reduce substrate availability for beneficial fermenters, and increase microbial dependence on endogenous host glycans. Responses nevertheless vary according to baseline microbiota, habitual diet, duration, and chemical composition of the administered oligosaccharide [4].

### 2.1. Mucus, Mucins, and the Intestinal Glycome

The effects of oligosaccharides must be considered within the glycan-rich interface separating the intestinal microbiota from the host epithelium. The intestinal glycome comprises glycans associated with secreted mucins, transmembrane mucins, epithelial glycoproteins, glycolipids, and proteoglycans. Together, the mucus layer and glycocalyx form a dynamic barrier in which glycans function as structural elements, microbial adhesion sites, soluble decoy receptors, immune ligands, and nutrient sources [3,7,8]. Mucins are heavily O-glycosylated proteins containing domains rich in proline, threonine, and serine. Their dense glycan decoration produces an extended bottle-brush conformation and enables gel formation and water retention. MUC2 (Mucin 2) is the principal gel-forming intestinal mucin, whereas transmembrane mucins contribute to the epithelial glycocalyx. Mucin-type O-GalNAc glycans commonly contain GalNAc (N-Acetylgalactosamine), galactose, GlcNAc (N-Acetylglucosamine), fucose, and sialic acid and may also be sulfated. Their terminal structures strongly influence microbial binding, resistance to enzymatic degradation, and interactions with endogenous lectins [8,9]. The mucus barrier is regionally organized. The small intestine is covered by a relatively penetrable mucus layer acting together with antimicrobial peptides, immunoglobulins, motility, and epithelial turnover. In the colon, a firmly adherent MUC2-rich inner layer normally limits bacterial access to epithelial cells, whereas the outer layer is colonized by the microbiota. Loss of MUC2 or defective O-glycosylation permits closer bacterial contact with the epithelium and increases inflammatory susceptibility, demonstrating that mucus function depends on both quantity and glycan composition [8,10].

### 2.2. Dietary Modulation of Mucins and the Intestinal Glycome

**Figure 1:**
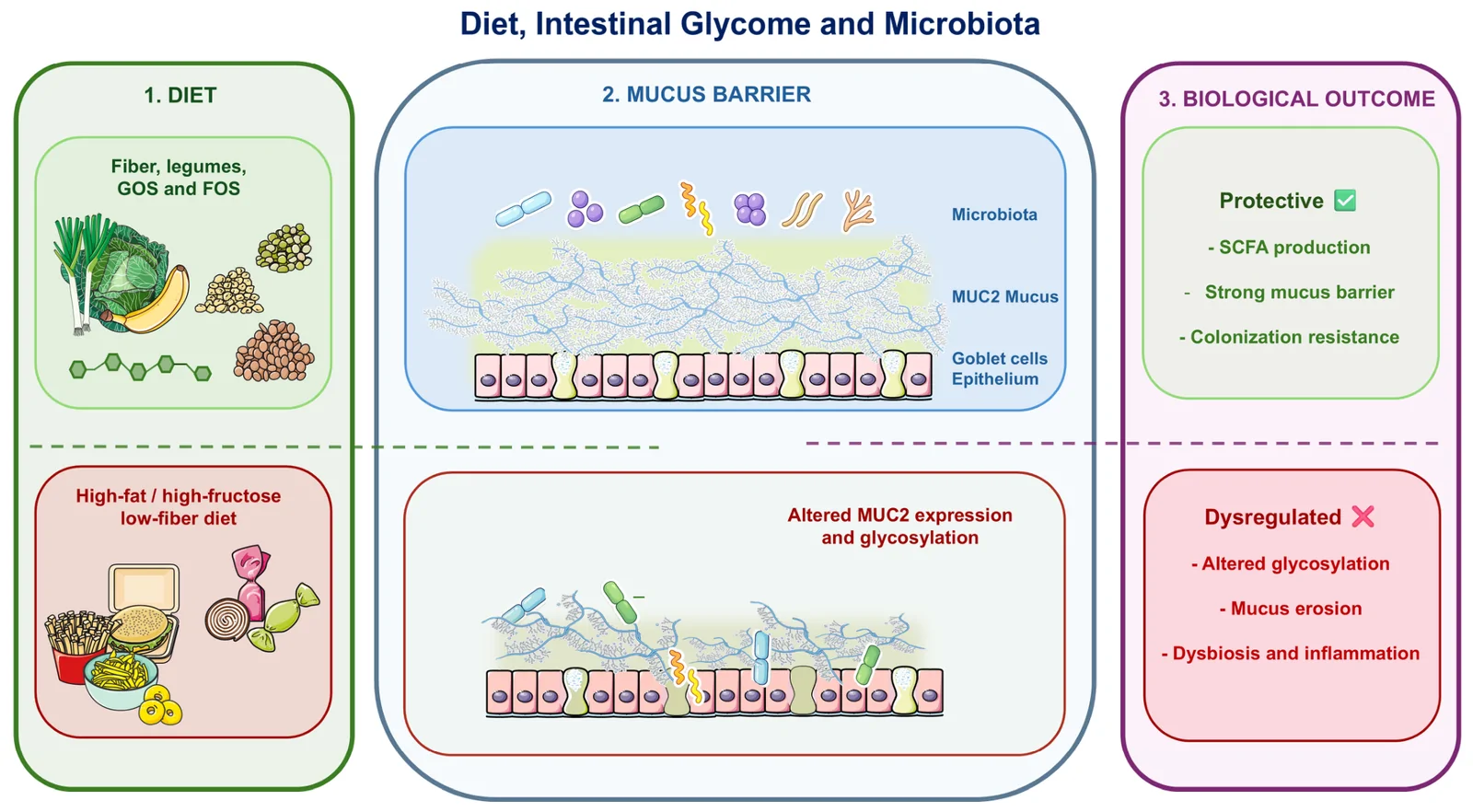
Dietary modulation of the intestinal mucus–glycome–microbiota axis. Fermentable oligosaccharides and fiber-rich foods support short-chain fatty acid production, goblet-cell function, mucin synthesis, and protective glycosylation.

The intestinal glycome is dynamic and responds to dietary, microbial, developmental, and inflammatory signals. Other dietary bioactive compounds, including polyphenols, may also influence intestinal inflammatory and metabolic signaling; however, these effects are beyond the main scope of this review [11]. Diet can influence glycosyltransferase and glycosidase expression, nucleotide-sugar availability, epithelial differentiation, mucin synthesis, and secretion. Microbial metabolites provide an additional regulatory layer, creating a bidirectional relationship in which diet modifies mucin glycans and the resulting glycan landscape shapes microbial adhesion, nutrient use, and spatial organization [3,7]. Experimental evidence illustrates the sensitivity of the mucus barrier to dietary composition. Prolonged high-fat feeding (42% fat, corresponding to 55% of total energy from fat, for 25 weeks) altered duodenal Brunner’s gland secretory activity while reducing fucosylated, sulfated, and terminal α1,4-GlcNAc-terminated glycans [12]. In the colon, high-fat feeding reduced Muc2 expression and altered mucin sialylation, sulfation, and the distribution of GlcNAc, GalNAc, mannose, galactose, and fucose. Supplementation with the chickpea accessions MG_13 and PI358934, incorporated as 10% raw crushed chickpea flour into the high-fat diet for 16 weeks, partially restored Muc2 expression and several oligosaccharide profiles, supporting a protective contribution of fermentable fiber and other food-derived bioactive compounds [10]. Supplementation with free fructose (30% *w*/*v* in drinking water for 16 weeks) provides a further example of age-dependent mucus remodeling in mice fed a high-fat diet providing 60% of total energy from fat. Younger animals displayed a mainly compensatory increase in selected mucin secretory and glycosylation markers, whereas adult animals showed reduced mucin production and marked losses of sialylation, GlcNAc, and fucosylation [13]. These preclinical findings suggest that age, duration of exposure, and epithelial plasticity influence the capacity of the mucosa to adapt to dietary stress. Dietary fiber availability also regulates microbial use of host mucus. Fiber deprivation can increase mucin foraging and erosion of the colonic mucus barrier, thereby enhancing pathogen susceptibility, whereas fermentable fiber or selected bifidobacteria can preserve mucus integrity [5,14]. Thus, dietary oligosaccharides may protect the host both by providing alternative microbial substrates and by supporting metabolite-mediated regulation of the epithelial barrier. The relationships among dietary composition, microbiota activity, mucus-barrier integrity, and the resulting biological outcomes are summarized in Figure 1.

In contrast, high-fat and high-fructose diets can alter MUC2 expression and mucin glycan composition, thereby weakening the mucus barrier and increasing microbial access to the epithelium. Several of the diet-induced changes in mucin expression and glycosylation illustrated here are based primarily on preclinical models and require confirmation in human studies.

## 3. Molecular Mechanisms Underlying Oligosaccharide-Mediated Modulation of Bacterial Colonization and Mucosal Host–Microbe Interactions

This section focuses on the molecular mechanisms by which oligosaccharides modulate bacterial adhesion, envelope integrity, host inflammatory responses, and biofilm development at the intestinal mucosa.

### 3.1. Interaction with the Bacterial Surface

The bacterial envelope is a hierarchically organized interface comprising LPS (lipopolysaccharides) or LOS (lipooligosaccharides), capsular and extracellular polysaccharides, teichoic acids, surface proteins, pili, fimbriae, and mucus-binding adhesins. The initial association of an oligosaccharide with a bacterium therefore often occurs at extracellular structures rather than through direct insertion into the cytoplasmic membrane. Bacterial adhesion proceeds through partially overlapping stages: weak electrostatic, hydrophobic, van der Waals, and glycan–glycan interactions bring the bacterium close to the mucosal surface, after which specific adhesin–glycan recognition stabilizes attachment [15]. Neutral HMOs, GOS, and related dietary glycans can occupy carbohydrate-recognition domains on bacterial adhesins, sterically mask the bacterial surface, modify adhesin organization, promote nonproductive aggregation, or reduce multivalent engagement. Such mechanisms can decrease adhesion without impairing viability. Manuka honey oligosaccharides, for example, reduced adhesion of *Escherichia coli* O157:H7, *Staphylococcus aureus*, and *Pseudomonas aeruginosa* to HT-29 cells without a corresponding bactericidal effect [16].

#### 3.1.1. Pili, Mucus-Binding Proteins, and Multivalent Adhesion

Pili extend glycan-binding domains beyond the crowded bacterial surface and help microorganisms overcome electrostatic and steric barriers. Their mechanical properties also influence retention under flow. In uropathogenic *E. coli*, type 1 pili terminate in the mannose-binding lectin FimH (Fimbrial adhesin H), whereas P pili carry PapG (P fimbrial adhesin G), which recognizes Galα1–4Gal. Uncoiling of the pilus shaft under tensile force reduces the load transmitted to the adhesin–receptor bond and can prolong attachment under physiological shear [15]. Multivalency converts individually weak interactions into high-avidity adhesion. *Lacticaseibacillus rhamnosus* GG (LGG), adhesins distributed along the pilus backbone mediate sequential mucin contacts and produce a zipper-like detachment pattern. Repeated domains in mucus-binding proteins can similarly display spring-like mechanical behavior. Under tensile force, sequential extension and detachment of individual adhesive domains can distribute the mechanical load across multiple contacts and increase the effective rupture distance, thereby helping to stabilize bacterial retention under shear. Studies using *Lactococcus lactis* mutants showed that both pili and mucus-binding proteins contribute to mucin adhesion, with compact proteins and extended pili producing distinct rupture-distance profiles. This mechanical behavior is relevant to anti-adhesive strategies because effective oligosaccharide inhibitors may need to disrupt multivalent adhesive interactions rather than a single receptor–ligand contact [15,17].

#### 3.1.2. Glycan–Glycan Interactions

Direct interactions between bacterial surface glycans and host glycans may also contribute to early attachment. Bacterial LPS, LOS (Lipo-oligosaccharide), capsules, and extracellular polysaccharides can recognize glycan structures displayed by mucins, glycoproteins, or glycolipids. Such interactions have been implicated in *Neisseria meningitidis* and *Shigella* adhesion. Individual glycan–glycan bonds are generally weak, but clustering and multivalency can generate biologically relevant avidity. Soluble oligosaccharides may therefore compete not only with protein adhesins but also with bacterial surface carbohydrates, although this mechanism remains less extensively characterized [15].

### 3.2. Direct Effects on Bacterial-Envelope Integrity

Direct membrane permeabilization is mechanistically distinct from anti-adhesive activity and should not be generalized to all oligosaccharides. Extensive evidence for charge-dependent antibacterial effects is available for polymeric chitosan [18,19], whereas evidence specifically obtained with structurally defined chitooligosaccharides is more limited. Protonation of amino groups in chitosan under acidic or mildly acidic conditions generates a positive charge that promotes electrostatic interactions with negatively charged bacterial-envelope components [18,19,20]. Selected chitooligosaccharides have also been reported to interact with bacterial envelopes and to increase membrane permeability and compromise membrane integrity in experimental bacterial models [20]. In Gram-negative bacteria, relevant targets include phosphate and carboxylate groups in LPS and anionic phospholipids. Chitosan binding can displace divalent cations, alter LPS packing, increase outer-membrane permeability, disturb the cytoplasmic membrane, dissipate ion gradients, and cause leakage of intracellular constituents. In Gram-positive bacteria, initial interactions involve teichoic and lipoteichoic acids before the underlying membrane is affected [18,19]. The antibacterial activity of chitosan and chitooligosaccharides is strongly influenced by molecular weight or degree of polymerization, degree of deacetylation, charge density, concentration, pH, ionic strength, and chemical derivatization; therefore, findings obtained with one chitosan-based compound should not be automatically extrapolated to another. This cationic mechanism should not be extrapolated to neutral FOS, GOS, or most HMOs, whose predominant effects involve microbial fermentation, host signaling, or interference with adhesion rather than nonspecific membrane lysis.

### 3.3. Targeted Inhibition of Bacterial-Envelope Metabolism

A distinct targeted mechanism is provided by the terminal α1,4-linked GlcNAc motif associated with gastric MUC6. This glycan inhibits the *H. pylori* cholesterol α-glucosyltransferase encoded by cgt/HP0421. Because *H. pylori* converts host cholesterol into cholesteryl glucosides that support membrane organization, colonization, and immune evasion, inhibition of this enzyme suppresses bacterial growth and compromises envelope-associated functions [21,22]. The intact branched glycan is more active than its isolated monosaccharide components, demonstrating that higher-order architecture is essential. This mechanism differs from both membrane lysis and receptor-decoy activity because it selectively targets bacterial-envelope biosynthesis.

### 3.4. Competition with Host–Cell Glycan Receptors

Soluble oligosaccharides act as decoy receptors when they reproduce structural motifs normally displayed by epithelial glycoproteins, glycolipids, or mucins. Bacterial adhesins bind the free glycan in the lumen or mucus rather than the membrane-anchored receptor. The resulting complexes lack the spatial organization needed for stable colonization and can be eliminated through mucus turnover, peristalsis, or epithelial shedding [15,21,23]. Anti-adhesive activity is highly structure dependent. Adhesins discriminate among anomeric configurations, linkage positions, neighboring residues, branching patterns, and terminal fucosylation or sialylation. Therefore, activity cannot be predicted from monosaccharide composition alone. Valency and ligand spacing are also crucial because a monovalent ligand may bind transiently yet fail to compete with a host surface displaying clustered receptors.

#### 3.4.1. HMOs and Enteric Pathogens

HMOs are especially suited to receptor mimicry because many contain fucosylated, sialylated, or lactosamine-based motifs resembling mucosal glycans. In the study by Manthey et al., preincubation of EPEC with pooled HMOs (10 mg/mL for 1 h) significantly reduced bacterial attachment to HeLa, HEp-2, and T84 epithelial cells, with a dose-dependent effect. In the same study, pooled HMOs also reduced intestinal EPEC colonization in suckling mice [22]. HMO fractions also inhibit adhesion of diarrheal pathogens, including *E. coli*, *Vibrio cholerae*, and *Salmonella*, to intestinal epithelial cells [21]. In addition to immediate decoy activity, HMOs can modify epithelial glycosylation and receptor presentation, indicating that acute competition and longer-term host glycome remodeling may occur simultaneously [2,21,22].

#### 3.4.2. GOS and Enteric Pathogens

GOS are heterogeneous mixtures of β-linked galactose-containing structures. Defined GOS preparations have reduced enteropathogenic *E. coli* adherence and *Salmonella typhimurium* adhesion, invasion, and infection in experimental systems [24,25]. However, in vivo reductions in pathogen burden may also reflect changes in microbiota composition, organic-acid production, goblet-cell activity, and barrier function. Direct binding assays and adhesin-deficient mutants are therefore required to demonstrate receptor mimicry as the dominant mechanism [26].

#### 3.4.3. FimH–Mannose and PapG–Galabiose Systems

Although the FimH–mannose and PapG–galabiose systems are primarily characterized in uropathogenic Escherichia coli and therefore represent extra-intestinal models, they provide well-defined examples of structure-specific adhesin–glycan recognition. FimH binds terminal α-D-mannose residues, and soluble mannosides can competitively occupy its binding pocket and reduce epithelial attachment. PapG recognizes the Galα1–4Gal epitope of globoseries glycolipids, and galabiose-containing oligosaccharides can act as receptor analogues. These systems illustrate how ligand structure, multivalency, and mechanical forces can influence anti-adhesive efficacy [15].

#### 3.4.4. BabA–Lewis b and SabA–Sialyl-Lewis Interactions

Although *Helicobacter pylori* colonizes the gastric rather than the intestinal mucosa and is not a primary focus of pediatric nutrition, this system provides a well-characterized mechanistic example of how inflammation-driven glycome remodeling and multivalent adhesin-glycan interactions can shape persistent mucosal colonization. *H. pylori* adapts its adhesive repertoire to the changing glycan landscape of the gastric mucosa. During the initial phases of colonization, the outer-membrane adhesin BabA (Blood group antigen-Binding Adhesin A) preferentially recognizes fucosylated ABO and Lewis b antigens displayed on gastric mucins and epithelial glycoconjugates [26,27,28,29]. This interaction promotes close contact with the gastric surface and contributes to the establishment of a stable mucosal niche. As infection persists, however, the inflammatory response progressively remodels epithelial glycosylation. In particular, the increased expression of sialylated Lewis antigens creates additional binding sites for the SabA (Sialic acid-Binding Adhesin) adhesin, enabling *H. pylori* to maintain attachment even when the original fucosylated receptor profile has been altered [30]. The transition from BabA-dominated to SabA-supported adhesion therefore illustrates how the bacterium exploits inflammation-associated changes in the host glycome rather than relying on a single, fixed receptor system Beyond glycan recognition, *H. pylori* also engages host proteins through interactions such as that between HopQ and carcinoembryonic antigen-related cell adhesion molecules, highlighting the diversity of its host-binding mechanisms [31].

### 3.5. Glycan Topology, Clustering, and Multivalency

Glycans on mucins and the epithelial glycocalyx are organized in heterogeneous patches rather than uniformly distributed. High local receptor density allows multiple bacterial adhesins to engage simultaneously, converting low-affinity monovalent contacts into high-avidity attachment. Longer or branched oligosaccharides, glycopolymers, and multivalent glycoconjugates may therefore outperform free monosaccharides by reproducing receptor clustering, engaging multiple lectin domains, or inducing nonproductive bacterial aggregation [15]. Greater valency is not automatically beneficial. Ligand spacing, linker flexibility, orientation, and accessibility determine whether a multivalent construct blocks adhesins, clusters them nonproductively, or inadvertently bridges bacteria to host surfaces. These parameters should be considered together with equilibrium affinity.

### 3.6. Modulation of Host Immune and Inflammatory Responses

In addition to acting on bacterial surfaces and host glycan receptors, oligosaccharides can modify infection outcomes by regulating epithelial and immune responses. Because oligosaccharides may support selected protective immune responses while simultaneously limiting excessive inflammatory signaling, we use the term “immune modulation” rather than nonspecific “immune activation” [32,33,34]. Their effects can be direct, through interactions with epithelial cells or glycan-binding immune receptors, or indirect, following microbial fermentation and the generation of SCFAs. Human milk oligosaccharides are the best-characterized immunomodulatory glycans. Selected HMOs can interact with host glycan-binding proteins, including selectins and specific C-type lectin receptors, and may thereby influence leukocyte adhesion or immune-cell signaling [34,35]. In intestinal epithelial models, HMOs and structurally related galactosyllactoses attenuated NF-κB-dependent inflammatory signaling, indicating that receptor mimicry may be accompanied by regulation of host–cell signaling [32]. Likewise, 2′-fucosyllactose reduced *Campylobacter jejuni*-associated epithelial inflammation and mucosal injury in experimental models, showing that anti-adhesive and anti-inflammatory effects can operate concurrently [33]. GOS and FOS often exert more indirect immunological effects. Their fermentation supports acetate, propionate, and butyrate production, which can reinforce tight junctions, regulate mucin secretion, and influence macrophage, dendritic-cell, and regulatory T-cell functions. GOS have also preserved epithelial barrier organization and reduced inflammatory responses in toxin-challenged intestinal models [36]. These findings indicate that lower cytokine concentrations after oligosaccharide supplementation do not necessarily demonstrate direct immune-receptor engagement; reduced bacterial contact, improved barrier integrity, and microbiota-derived metabolites may be equally important. Immunomodulatory outcomes remain strongly dependent on glycan structure, purity, dose, developmental stage, inflammatory status, and baseline microbiota. Epithelial monocultures, organoids, and animal models each capture different components of mucosal biology but cannot fully reproduce the complexity of the human intestinal ecosystem. Findings from these experimental systems should therefore be interpreted as mechanistic or translational evidence and should not be directly extrapolated to clinical outcomes without validation in human studies. Overall, current data support a role for oligosaccharides in balancing mucosal defense and inflammatory tone rather than functioning as nonspecific immune stimulants.

### 3.7. Effects on Bacterial Biofilm Formation

Biofilm formation is a multistage process comprising reversible attachment, irreversible adhesion, microcolony development, extracellular-matrix production, maturation, and dispersal. Oligosaccharides may interfere at different stages, but anti-adhesive, biofilm-preventive, biofilm-disrupting, and bactericidal activities should not be treated as equivalent. A compound that reduces initial attachment may prevent biofilm establishment without eliminating an already mature community. By occupying adhesins or mimicking host glycans, neutral oligosaccharides can reduce the initial surface contacts required for microcolony formation. They may also alter bacterial aggregation, motility, cell-surface properties, or the synthesis and organization of extracellular polymeric substances. HMOs provide a representative example: defined HMO fractions inhibited growth and biofilm formation by Group B Streptococcus, with activity varying across glycan fractions, indicating that the effect depends on specific structural features rather than on total carbohydrate content alone [37,38]. Interpretation nevertheless requires complementary measurements, because reduced biofilm biomass may reflect impaired adhesion, lower bacterial growth, altered matrix production, or combinations of these effects.

Polymeric chitosan, chemically modified chitosans, and selected chitooligosaccharides can interfere with bacterial adhesion and biofilm development through charge-dependent mechanisms, although the available evidence differs substantially among these compound classes. Their activity is influenced by molecular weight or degree of polymerization, degree of deacetylation, pH, solubility, and charge density. For example, sulfonated chitosan has been shown to inhibit Pseudomonas aeruginosa adhesion and biofilm development, illustrating how chemical modification of chitosan can influence antibiofilm activity [39]. The effects of GOS, FOS, and plant-derived oligosaccharides are more context-dependent. A non-utilizable glycan may inhibit attachment, whereas a fermentable substrate can support growth or surface persistence of organisms able to metabolize it. Promotion of biofilm formation is not necessarily detrimental when it enhances mucosal retention of beneficial lactobacilli or bifidobacteria, but it may be undesirable for opportunistic pathogens. Studies should therefore assess pathogenic and commensal species separately and evaluate initial adhesion, mature-biofilm viability, matrix composition, and planktonic growth as distinct endpoints.

### 3.8. Mechanistic Diversity of Oligosaccharide-Mediated Effects

**Figure 2:**
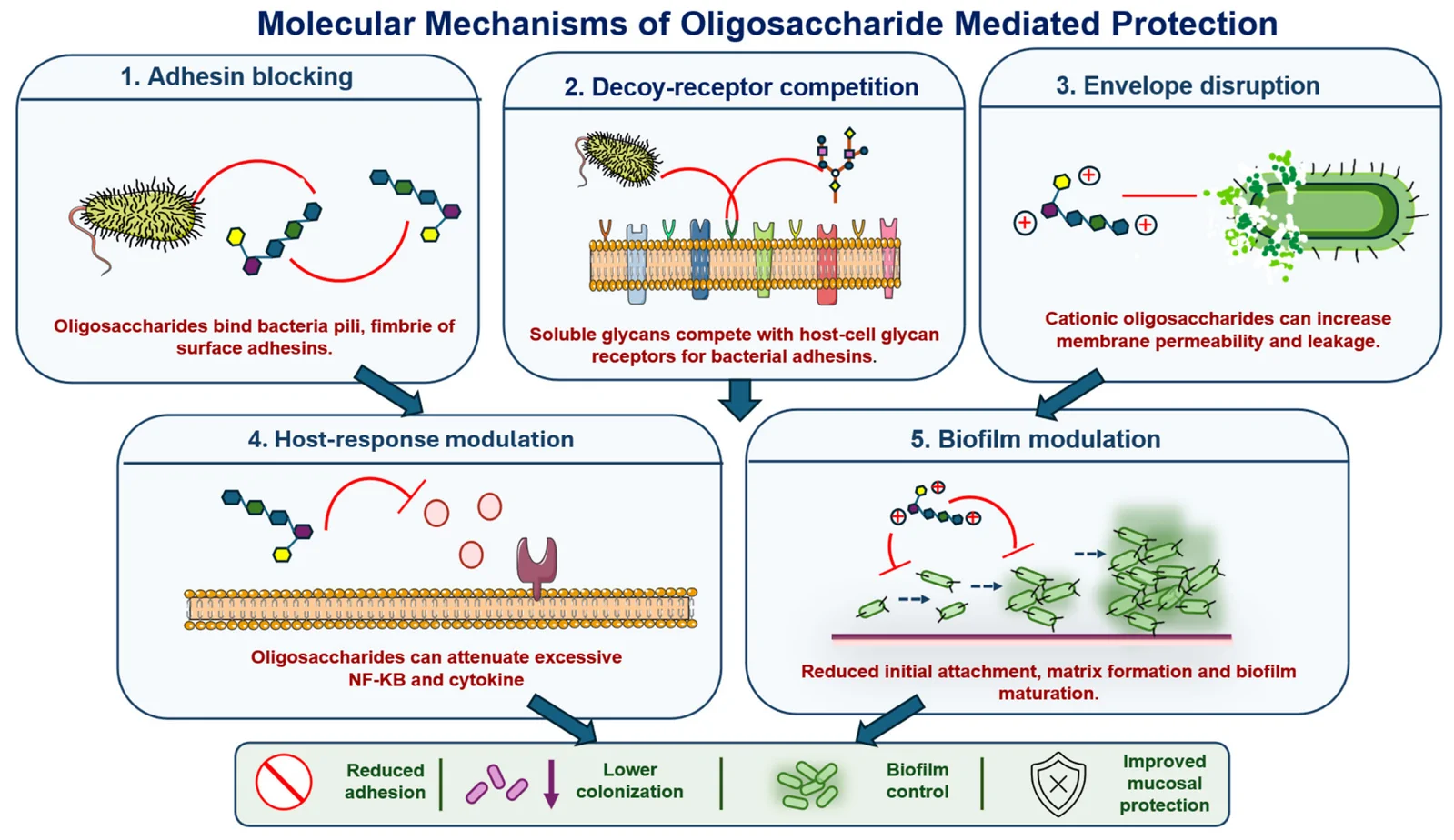
Molecular mechanisms by which oligosaccharides interfere with bacterial colonization. Neutral oligosaccharides mainly act as soluble decoy receptors and ligands for bacterial adhesins, whereas cationic oligosaccharides can additionally destabilize bacterial envelopes. Oligosaccharides may also modulate epithelial and immune signaling and interfere with biofilm initiation and maturation. The mechanisms illustrated are structure- and compound-dependent and should not be interpreted as shared properties of all oligosaccharides.

Oligosaccharides can reduce bacterial colonization through multiple, often overlapping mechanisms, including adhesin binding, competition with host glycans, modulation of epithelial glycosylation, microbiota-mediated colonization resistance, and direct effects on the bacterial envelope [2,15,18,22,25,31]. Consequently, reduced adhesion or viability should not be interpreted as evidence of a single mechanism. Neutral HMOs and GOS mainly act through glycan mimicry, receptor competition, and host- or microbiota-mediated pathways [21,22,24,25], whereas direct membrane damage is best established for polymeric chitosan [19,20], with more limited evidence supporting membrane-disruptive effects of selected chitooligosaccharides. α1,4-GlcNAc-terminated MUC6 glycans instead illustrate a more selective inhibition of bacterial-envelope metabolism in *Helicobacter pylori* [40,41]. Recognizing these mechanistic differences is essential for the accurate interpretation and therapeutic development of oligosaccharide-based interventions. The principal direct and indirect mechanisms through which oligosaccharides may limit bacterial colonization and support mucosal protection are summarized in Figure 2.

A comparative overview of the main oligosaccharide and glycan classes, their structural features, predominant mechanisms, representative evidence, and potential pediatric relevance is provided in Table 1.

## 4. Oligosaccharides in Pediatric Health: Effects on Gut Microbiota and Immune Function

The molecular mechanisms described above acquire particular clinical relevance during infancy and childhood, when the intestinal microbiota, mucus barrier, epithelial glycome, and immune system are still undergoing maturation. Pediatric populations therefore provide an important context in which the microbiota-mediated, anti-adhesive, barrier-supporting, and immunomodulatory effects of oligosaccharides may influence both short- and long-term health outcomes.

### 4.1. Human Milk Oligosaccharides and Early Microbial Colonization

Human milk is a complex biological system containing nutrients and bioactive molecules that contribute to infant growth and development. Omics-based studies have highlighted the coordinated biological activities of its proteins, lipids, metabolites, microorganisms, and glycans, as well as their potential contribution to systemic functions such as immune, metabolic, and bone homeostasis [42]. Within this complex network, human milk oligosaccharides represent one of the most abundant and biologically active groups of glycans.

The neonatal period represents a critical developmental window for the maturation of the intestinal microbiome. Alterations in microbial colonization during this period have been associated with an increased susceptibility to infections, food allergy, and immune-mediated diseases later in life [43].

Early microbial colonization is influenced by multiple factors, including mode of delivery, gestational age, prenatal and postnatal antibiotic exposure, skin-to-skin contact, and infant feeding [44].

Breastfed infants typically develop a microbiota enriched in *Bifidobacterium* species, whereas formula-fed infants generally show a more diverse microbial profile, often characterized by lower relative abundances of bifidobacteria and higher abundances of selected members of the Enterobacteriaceae, Clostridiaceae, and Enterococcaceae families [45]. These differences in early microbial composition have been associated with susceptibility to infectious and inflammatory diseases later in life, although the underlying relationships remain multifactorial.

One of the principal factors responsible for the characteristic protective microbiota observed in breastfed infants is the high concentration of human milk oligosaccharides (HMOs), which constitute the third largest solid component of human milk after lactose and lipids [46]. Their concentration is highest during the first days after delivery and gradually declines throughout lactation, accounting for approximately 5–15 g/L in mature milk and 20–25 g/L in colostrum. Because HMOs are highly resistant to digestion in the upper gastrointestinal tract, they reach the colon largely intact. Their selective utilization by members of the gut microbiota, particularly selected *Bifidobacterium* species, primarily promotes acetate and lactate production. These metabolites may also support propionate- and butyrate-producing microorganisms through microbial cross-feeding. The resulting metabolic activity can contribute to intestinal homeostasis by lowering luminal pH, supporting epithelial barrier integrity and mucus production, and modulating local immune responses [47].

These effects may be particularly relevant in preterm infants, whose immature intestinal barrier, delayed initiation of enteral nutrition, frequent exposure to antibiotics and oxygen therapy, and increased susceptibility to infection contribute to the risk of severe gastrointestinal complications, including necrotizing enterocolitis (NEC) [48]. Masi et al. reported an observational association between lower DSLNT concentrations in maternal milk and subsequent NEC in preterm infants. These differences were accompanied by distinct microbial patterns before NEC onset, including a lower relative abundance of *Bifidobacterium* and a higher abundance of *Enterobacter* [49]. Although these findings support a potential relationship between HMO composition, microbial ecology, and NEC susceptibility, they do not establish that DSLNT or HMO supplementation prevents NEC. Current international guidelines recommend mother’s own milk as the preferred source of enteral nutrition for preterm or low-birth-weight infants [50].

### 4.2. GOS- and FOS-Supplemented Infant Formulas

However, human milk is not always available or sufficient. This limitation has contributed to the development of infant formulas supplemented with fermentable oligosaccharides, primarily galacto-oligosaccharides (GOS) and fructo-oligosaccharides (FOS), with the aim of reproducing some of the microbiota-modulating effects associated with HMOs. Nevertheless, GOS and FOS are structurally less complex than HMOs and cannot reproduce all their biological functions.

GOS and FOS are non-digestible carbohydrates with distinct structures and production processes. GOS are generally produced from lactose through the transgalactosylation activity of β-galactosidase, whereas short-chain FOS are commonly synthesized from sucrose by fructosyltransferases; longer-chain fructans may also be obtained through the controlled hydrolysis of inulin. Their selective utilization by intestinal microorganisms can modify microbial composition and metabolic activity, including the production of SCFAs. These effects depend on oligosaccharide structure, dose, and the baseline composition of the gut microbiota.

Beyond their effects on intestinal physiology, the prebiotic activity of GOS and FOS indirectly contributes to protection against bacterial infections by increasing colonization resistance. The expansion of beneficial commensals limits pathogen growth through competition for nutrients and epithelial adhesion sites, production of antimicrobial metabolites, and modulation of host immune responses.

Recent microbiome studies support the microbiota-modulating effects of GOS, although these findings should not by themselves be interpreted as evidence of protection against infection. In a randomized controlled intervention involving 210 healthy infants, Heppner et al. showed that GOS-containing milk formula sustained high levels of *Bifidobacterium* during the first year of life. Interestingly, GOS supplementation also influenced the development of diurnal oscillations in microbial taxa and metabolic pathways, with patterns resembling those observed in breastfed infants [51].

A 2025 systematic review and meta-analysis specifically evaluating formulas supplemented with scGOS/lcFOS in a 9:1 ratio included 18 publications comprising 1675 infants and young children, with 11 studies included in the quantitative analysis. Overall, scGOS/lcFOS-supplemented formulas were well tolerated and were associated with beneficial changes in gastrointestinal parameters and markers related to intestinal microbiota and immunity. The available evidence was particularly supportive of effects on stool characteristics, intestinal environment and beneficial bacterial colonization, whereas evidence for prevention of infectious or allergic diseases was less uniform [52].

These findings are consistent with the most recent recommendations of the European Society for Paediatric Gastroenterology, Hepatology and Nutrition (ESPGHAN). The authors concluded that the biotic-supplemented formulas evaluated to date do not raise major safety concerns regarding growth, tolerance or adverse effects in healthy infants. However, for scGOS/lcFOS, only a weak recommendation in favor of supplementation could be formulated. The most consistently demonstrated clinical effect was softer stool consistency, with a smaller effect on stool frequency [53].

Another important consideration is that recent studies increasingly evaluate complex formulations containing combinations of prebiotics, probiotics, postbiotics or other bioactive components rather than isolated GOS/FOS. Although such approaches may more closely reproduce the complexity of human milk, they make it difficult to determine the individual contribution of each component.

### 4.3. Oligosaccharides and Antibiotic-Associated Dysbiosis

During childhood, another potential application of prebiotic oligosaccharides is the prevention or attenuation of antibiotic-induced gut dysbiosis. Antibiotic exposure can profoundly disrupt the developing intestinal microbiota, reducing beneficial bacterial populations and microbial metabolic activity while favoring the expansion of potentially pathogenic taxa, especially during prolonged antibiotic therapy [54,55].

At the mechanistic and preclinical level, in vitro studies suggest that selected oligosaccharides may support microbiota recovery following antibiotic exposure, although these experimental findings require confirmation in clinical studies [56].

Using the dynamic TIM-2 in vitro colon model inoculated with fecal microbiota from breastfed infants, Endika et al. showed that exposure to amoxicillin/clavulanate markedly altered microbial composition and activity, causing an expansion of enterobacteria and a decrease in short-chain fatty acid production [56].

Interestingly, supplementation with 2′-fucosyllactose (2′-FL) or GOS supported microbiota recovery. In particular, 2′-FL promoted recovery and restored propionate and butyrate production in microbiota derived from 1-month-old infants, whereas GOS increased acetate and butyrate production and supported recovery in the more *Bifidobacterium*-dominated microbiota of 3-month-old infants [56].

When the model was exposed to a different class of antibiotics, such as the macrolide azithromycin, the effect of GOS was less pronounced. GOS supplementation did not promote recovery of the original microbial community composition within the 72-h experimental period, although it continued to enhance microbial metabolic activity, including SCFA production [57]. Although these experimental findings provide a biological rationale for oligosaccharide supplementation during antibiotic therapy, they cannot by themselves establish a clinical preventive effect. Beyond their local intestinal effects, SCFAs can enter the portal and systemic circulation and act as signaling metabolites, including through G protein-coupled receptors and histone deacetylase-dependent mechanisms, thereby influencing metabolic and immune functions at extra-intestinal sites such as the liver, lungs, and brain [58].

At the observational level, clinical evidence in children remains heterogeneous, these experimental findings provide a biological rationale for oligosaccharide supplementation during antibiotic therapy, clinical evidence in children is heterogeneous across available studies. Nevertheless, clinical observations suggest that oligosaccharides may contribute to shaping the gut microbiota during antibiotic exposure, although their effects appear to vary according to feeding modality, oligosaccharide composition and the type of antibiotic used. In a large cohort of one-month-old infants in which the impact of early antibiotic exposure was specifically investigated, breastfeeding was strongly associated with a *Bifidobacterium*-rich microbiota, and specific breast milk HMOs, particularly lacto-N-hexaose (LNH), were positively associated with the relative abundance of *Bifidobacterium*. Among exclusively formula-fed infants, GOS-supplemented formula was instead associated with a lower relative abundance of *Clostridium perfringens* [59]. In a randomized placebo-controlled trial involving 258 children aged 3–6 years, Soldi et al. investigated the effects of supplementation with 6 g/day of inulin-type fructans over a 24-week period, including in children who required antibiotic treatment during the study. As expected, antibiotic exposure reduced the relative abundance of *Bifidobacterium* in both intervention and control groups. However, children receiving prebiotic supplementation maintained significantly higher levels of *Bifidobacterium* at the end of antibiotic treatment compared with those receiving placebo [60]. The ongoing TOBBI randomized controlled trial is designed to determine whether a six-week nutritional intervention initiated on the last day of antibiotic treatment can accelerate gut microbiota recovery in toddlers aged 12–36 months treated with amoxicillin or amoxicillin/clavulanate. The intervention consists of a cow’s-milk-based formula supplemented with short-chain GOS and long-chain FOS (scGOS/lcFOS, 9:1), together with *Bifidobacterium* breve M-16V [61]. Because this was a multicomponent intervention, however, the specific contribution of the oligosaccharide component cannot be clearly established. Overall, the available evidence suggests that prebiotic oligosaccharides may help preserve selected microbial populations or metabolic functions during and after antibiotic exposure. However, most evidence is derived from in vitro models, observational studies, or multicomponent interventions, and their ability to accelerate microbiota recovery in children has not yet been established. More broadly, evidence for microbiota modulation should not be equated with evidence of clinically relevant disease prevention, which remains limited.

### 4.4. Clinical Outcomes and Current Limitations

The biological effects of HMOs may extend beyond their immediate influence on the infant gut microbiota, with possible longer-term associations with susceptibility to infection and antibiotic exposure. Berger et al. [62] analyzed microbiota data obtained from a randomized trial of an infant formula supplemented with 2′-FL and LNnT. Although the nutritional intervention was randomized, the reported relationship between fecal community type at 3 months and subsequent antibiotic use was an observational association and should not be interpreted as evidence that a specific microbial profile causally reduced antibiotic requirements [62]. The possible effects of prebiotic oligosaccharides have also been investigated beyond infancy. Some pediatric nutritional products contain GOS or FOS together with probiotics and other bioactive ingredients and are intended to support immune function. However, because these are multicomponent formulations, their clinical effects cannot be attributed specifically to the oligosaccharide component. A randomized pediatric study of a multistrain synbiotic formulation containing prebiotic components reported reductions in selected respiratory infection outcomes. However, because the intervention was multicomponent, the independent contribution of the oligosaccharide component cannot be determined [63]. Current evidence supports the central role of HMOs in shaping the infant gut microbiota and indicates that supplemented oligosaccharides such as GOS and FOS can influence microbial composition, fermentation activity, and stool characteristics. Evidence for the prevention of infectious, allergic, or inflammatory diseases is less consistent. Further clinical studies should evaluate structurally defined oligosaccharides as stand-alone interventions and use clinically relevant endpoints to determine their independent contribution to microbiota recovery, immune function, and pediatric health.

## 5. Conclusions

Oligosaccharides comprise a structurally heterogeneous group of glycans whose effects on intestinal homeostasis extend beyond conventional prebiotic activity. Depending on their structure, they can influence microbial fermentation and cross-feeding, mucosal glycosylation and barrier function, bacterial adhesion, immune signaling, and, for selected compounds, bacterial growth or envelope integrity. These mechanisms are compound-specific and should not be generalized across chemically distinct oligosaccharides. In early life, HMOs have a well-established role in shaping the infant gut microbiota, while GOS- and FOS-containing formulas can reproduce selected microbiota-, metabolite-, and stool-related effects. However, changes in microbial composition or metabolism should not be equated with prevention of specific pediatric diseases. Evidence for protection against infectious, allergic, inflammatory, or antibiotic-associated outcomes remains heterogeneous, and associations between individual HMO profiles and conditions such as NEC do not establish a preventive effect of supplementation. Future studies should evaluate structurally defined compounds, distinguish mechanistic from clinical endpoints, and prioritize well-designed pediatric trials capable of establishing causal clinical benefit.

## Figures and Tables

**Table 1:** Structure–function relationships, representative evidence, and potential pediatric relevance of selected oligosaccharides and glycans.

Oligosaccharide	Relevant Structural Feature(s)	Main Molecular/Biological Mechanism(s)	Representative Evidence/Level of Evidence	Potential Pediatric Relevance
(HMOs)	Structurally diverse glycans built on a lactose core; may be neutral, fucosylated, or sialylated.	Selective utilization by bifidobacteria, cross-feeding and SCFA production; receptor mimicry and anti-adhesive activity; modulation of epithelial glycosylation, barrier function, and immune signaling.	Anti-adhesion has been shown in epithelial and animal models; immunomodulatory effects are supported by cellular/preclinical studies; human studies link HMO exposure to early-life microbiota profiles. Level: preclinical, observational, and intervention-derived evidence.	Central to early-life microbiota development and may support intestinal and immune maturation. Evidence is strongest for microbiota/metabolite outcomes; disease-prevention effects remain heterogeneous.
2′-Fucosyllactose (2′-FL)	Fucosylated HMO containing a terminal fucose residue.	Receptor mimicry and anti-adhesive activity; modulation of epithelial inflammatory signaling; support of microbial metabolic recovery after antibiotic exposure.	Reduced *Campylobacter*-associated inflammation in experimental models and supported microbiota and SCFA recovery after amoxicillin/clavulanate in an infant-derived in vitro model. A randomized infant-formula trial used both 2′-FL and LNnT; the association between fecal community type and later antibiotic use was observational and cannot be attributed to 2′-FL alone. Level: preclinical/in vitro and clinical observational evidence.	Biologically plausible role in mucosal protection and microbiota recovery, but independent pediatric efficacy for prevention of infection or other clinical outcomes remains unestablished.
(GOS)	Heterogeneous β-linked galactose-containing oligosaccharides, commonly produced from lactose.	Selective fermentation; promotion of bifidobacteria and SCFA production; colonization resistance; anti-adhesive effects; support of epithelial barrier function.	Reduced enteropathogenic *E. coli* adherence and *Salmonella* infection in experimental systems and supported epithelial barrier responses. GOS-containing formula maintained high *Bifidobacterium* abundance in infants. Level: preclinical and randomized clinical evidence.	Widely used in infant formula. Effects on microbiota and gastrointestinal parameters are relatively consistent; evidence for prevention of infectious, allergic, or inflammatory disease is less consistent.
FOS/scGOS–lcFOS	Short-chain fructose-containing glycans; infant formulas may contain scGOS/lcFOS mixtures, commonly in a 9:1 ratio.	Fermentation and SCFA production; promotion of beneficial microbial populations; indirect enhancement of colonization resistance and barrier function.	Meta-analysis of scGOS/lcFOS formulas reported favorable gastrointestinal and microbiota-related outcomes; ESPGHAN issued only a weak recommendation. Inulin-type fructans preserved *Bifidobacterium* during antibiotic treatment; TOBBI currently provides protocol, not outcome, data. Level: clinical trials/evidence synthesis plus ongoing research.	May improve stool characteristics and selected microbiota-related outcomes. Evidence for prevention of infectious, allergic, or inflammatory disease remains heterogeneous.
Chitosan-based compounds	Cationic glucosamine-rich compounds; activity depends on molecular weight/degree of polymerization, deacetylation, pH, charge density, and derivatization.	Charge-dependent interaction with bacterial envelopes; increased membrane permeability and impaired integrity; interference with adhesion and biofilm architecture in selected systems.	Polymeric chitosan has established charge-dependent antibacterial effects. Selected chitooligosaccharides can increase membrane permeability, whereas sulfonated chitosan inhibits *Pseudomonas aeruginosa* adhesion and biofilm development. Level: predominantly in vitro/preclinical.	Illustrates direct antimicrobial and antibiofilm mechanisms distinct from conventional prebiotic effects. Direct pediatric clinical relevance has not been established.
MUC6 α1,4-GlcNAc glycans	Terminal α1,4-linked GlcNAc motif associated with gastric MUC6; activity depends on intact glycan architecture.	Selective inhibition of *Helicobacter pylori* cholesterol α-glucosyltransferase, impairing cholesteryl-glucoside synthesis and suppressing bacterial growth.	Mechanistic studies demonstrate inhibition of *H. pylori* cholesterol α-glucosyltransferase and the natural antibacterial function of gastric mucin. Level: mechanistic/preclinical.	Provides a structure–function paradigm for glycan-mediated inhibition of bacterial metabolism. Direct relevance to pediatric intestinal nutrition is limited.
Mannosides and galabiose glycans	Terminal mannose is recognized by FimH; Galα1–4Gal motifs are recognized by PapG. These systems are mainly characterized in uropathogenic *E. coli*.	Competitive occupancy of adhesin-binding pockets and inhibition of stable host–cell attachment; efficacy is influenced by ligand structure, multivalency, and mechanical forces.	FimH–mannose and PapG–galabiose systems are well-characterized models of structure-specific, force-dependent anti-adhesive interactions. Level: mechanistic, mainly extra-intestinal.	Useful model systems for rational anti-adhesive glycan design, but direct pediatric gastrointestinal evidence is currently limited.

Abbreviations: 2′-FL, 2′-fucosyllactose; FOS, fructo-oligosaccharides; GOS, galacto-oligosaccharides; HMOs, human milk oligosaccharides; lcFOS, long-chain fructo-oligosaccharides; MUC6, mucin 6; SCFA, short-chain fatty acids; scGOS, short-chain galacto-oligosaccharides.

## Data Availability

No new data were created or analyzed in this study. Data sharing is not applicable to this article.

## Abbreviations

2′-FL	2′-Fucosyllactose
BabA	Blood group antigen-binding adhesin A
FimH	Fimbrial adhesin H
FOS	Fructo-oligosaccharides
GOS	Galacto-oligosaccharides
HMOs	Human milk oligosaccharides
lcFOS	Long-chain fructo-oligosaccharides
LGG	Lacticaseibacillus rhamnosus GG
LNH	Lacto-N-hexaose
LOS	Lipooligosaccharide
LPS	Lipopolysaccharide
MUC2	Mucin 2
MUC6	Mucin 6
GalNAc	N-Acetylgalactosamine
GlcNAc	N-Acetylglucosamine
NF-κB	Nuclear factor kappa B
PapG	P fimbrial adhesin G
SabA	Sialic acid-binding adhesin
SCFAs	Short-chain fatty acids
scGOS	Short-chain galacto-oligosaccharides
